# Optimization of cDNA microarrays procedures using criteria that do not rely on external standards

**DOI:** 10.1186/1471-2164-8-377

**Published:** 2007-10-18

**Authors:** Torunn Bruland, Endre Anderssen, Berit Doseth, Hallgeir Bergum, Vidar Beisvag, Astrid Lægreid

**Affiliations:** 1 Department of Cancer Research and Molecular Medicine, Faculty of Medicine, Norwegian University of Science and Technology (NTNU), N-7489 Trondheim, Norway.; 2 NTNU Microarray Core Facility, Norwegian University of Science and Technology (NTNU), N-7489 Trondheim, Norway.

## Abstract

**Background:**

The measurement of gene expression using microarray technology is a complicated process in which a large number of factors can be varied. Due to the lack of standard calibration samples such as are used in traditional chemical analysis it may be a problem to evaluate whether changes done to the microarray procedure actually improve the identification of truly differentially expressed genes. The purpose of the present work is to report the optimization of several steps in the microarray process both in laboratory practices and in data processing using criteria that do not rely on external standards.

**Results:**

We performed a cDNA microarry experiment including RNA from samples with high expected differential gene expression termed "high contrasts" (rat cell lines AR42J and NRK52E) compared to self-self hybridization, and optimized a pipeline to maximize the number of genes found to be differentially expressed in the "high contrasts" RNA samples by estimating the false discovery rate (FDR) using a null distribution obtained from the self-self experiment. The proposed high-contrast *versus *self-self method (HCSSM) requires only four microarrays per evaluation. The effects of blocking reagent dose, filtering, and background corrections methodologies were investigated. In our experiments a dose of 250 ng LNA (locked nucleic acid) dT blocker, no background correction and weight based filtering gave the largest number of differentially expressed genes. The choice of background correction method had a stronger impact on the estimated number of differentially expressed genes than the choice of filtering method. Cross platform microarray (Illumina) analysis was used to validate that the increase in the number of differentially expressed genes found by HCSSM was real.

**Conclusion:**

The results show that HCSSM can be a useful and simple approach to optimize microarray procedures without including external standards. Our optimizing method is highly applicable to both long oligo-probe microarrays which have become commonly used for well characterized organisms such as man, mouse and rat, as well as to cDNA microarrays which are still of importance for organisms with incomplete genome sequence information such as many bacteria, plants and fish.

## Background

Gene-expression microarrays are widely used for large scale studies in cells, and are emerging as promising tools in clinical diagnosis, with potential impact on the assessment of prognosis and choice of treatment [1]. Microarray data are also increasingly becoming essential for data driven model building in system biology approaches [2,3]. However, the measurement of gene expression using e.g. cDNA microarray technology is a complex process that involves several steps, which have not yet been fully optimized. In a typical cDNA microarray experiment, total RNA from two biological sources in which gene expression levels are to be compared, is labelled with two different fluorescent dyes and hybridized to an array of spotted cDNA probes. After hybridization and scanning the data are further pre-processed. This often includes image analysis, filtering of data and background subtraction before normalization and transformation of data. Relative gene expression levels are given as ratios of intensities of fluorescent emission from labelled the RNAs'. Many different approaches and methods can be used at each stage of a microarray experiment, and there is still no definitive consensus about which methods to choose (reviewed and discussed in [4]).

Sample quality, labeling protocol, hybridization conditions, scanning protocols and image acquisition as well as the different stages in the microarray data analysis pipeline can all contribute to the overall uncertainty of the conclusions drawn. There has therefore recently been an emerging focus on the need for universally applicable standards, reference materials and analytic guidelines to assist in the standardization of microarray experiments [4-10]. In order to choose or optimize microarray methods several criteria can be used. The use of external RNA spike-in controls is the most recommended approach for technology assessment and optimization. However, the recommended use of spike-in RNA might not always be feasible. For optimal application several controls and probes must be used and the spike-in controls must be representative of the endogenous RNA with respect to e.g. length, sequence characteristics, melting temperature and cross-hybridization risk [7]. On some arrays suitable spike-in controls are not printed, and printing a sufficient number of spikes can leave insufficient room for the genes of biological interest. Other valuable tools are calibrated reference RNA samples such as those developed in the MAQC project [10] or mixed tissue RNA samples developed by Thompson et al. [11,12]. However, it is not always possible to have RNA samples with known differences in the expression level of many genes from the species of interest.

One general strategy that can be used is to minimize the variability of self-self hybridizations [13]. Unfortunately, this criterion focuses on the zero ratios that are the least interesting biologically and will tend to minimize variability (noise) as well as signal, which may not improve the overall ability to detect differentially expressed genes. One major concern is that the optimization criterion should increase the chances of discovering truly differentially expressed genes. Here we propose an optimization method that requires only four microarrays per evaluation and uses both hybridizations of RNA from samples with marked differences in gene expression, termed "high contrasts" as well as self-self hybridizations. A change in either laboratory procedure or data processing can increase real signal or just increase the noise. If the noise is increased this will also affect the self-self hybridizations. On the other hand, if signal is lost and genes are pushed artificially towards zero ratios, this will shrink ratios in the high contrast experiments. In the high contrast *versus *self-self method (HCSSM) proposed here we maximize the difference between a self-self and a high contrast experiment. This difference is quantified as the number of significantly differentially expressed genes in the high contrast experiment at a certain false discovery rate (FDR) estimated using the self-self experiments as a null distribution. Genes are scored for differential expression on the basis of the high contrast and the self-self hybridization separately using a modified T- statistic [14].

t˜g=β^gs˜gνg
 MathType@MTEF@5@5@+=feaafiart1ev1aaatCvAUfKttLearuWrP9MDH5MBPbIqV92AaeXatLxBI9gBaebbnrfifHhDYfgasaacH8akY=wiFfYdH8Gipec8Eeeu0xXdbba9frFj0=OqFfea0dXdd9vqai=hGuQ8kuc9pgc9s8qqaq=dirpe0xb9q8qiLsFr0=vr0=vr0dc8meaabaqaciaacaGaaeqabaqabeGadaaakeaacuWG0baDgaacamaaBaaaleaacqqGNbWzaeqaaOGaeyypa0ZaaSaaaeaaiiGacuWFYoGygaqcamaaBaaaleaacqqGNbWzaeqaaaGcbaGafm4CamNbaGaadaWgaaWcbaGaee4zaCgabeaakmaakaaabaGae8xVd42aaSbaaSqaaiabbEgaNbqabaaabeaaaaaaaa@3A5D@

**Figure 1:**
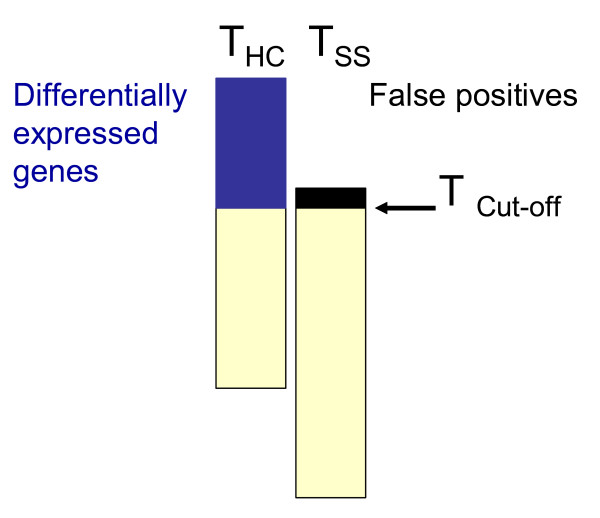
**The High Contrast *versus *Self-Self method (HCSSM)**. The figure illustrates how the false discovery rate is determined in HCSSM. For a chosen T cut-off, the genes with T-scores larger than the cut-off are declared significant. The false discovery rate is then determined by dividing the number of genes deemed significant in the self-self experiment by the number of genes deemed significant in the high contrast experiment. For example, if for a T-cut-off six genes are declared significant from the self-self experiment and 127 genes are declared significant from the high contrast experiment the false discovery rate will be 6/127 ~0.05. If a specific false discovery rate is wanted (often 0.05 or 0.01), the T cut-off can be adjusted to obtain it.

Where β^g
 MathType@MTEF@5@5@+=feaafiart1ev1aqatCvAUfKttLearuWrP9MDH5MBPbIqV92AaeXatLxBI9gBaebbnrfifHhDYfgasaacH8akY=wiFfYdH8Gipec8Eeeu0xXdbba9frFj0=OqFfea0dXdd9vqai=hGuQ8kuc9pgc9s8qqaq=dirpe0xb9q8qiLsFr0=vr0=vr0dc8meaabaqaciaacaGaaeqabaqabeGadaaakeaaiiGacuWFYoGygaqcamaaBaaaleaacqqGNbWzaeqaaaaa@2FE6@ is the estimated effect for gene *g*, s˜g
 MathType@MTEF@5@5@+=feaafiart1ev1aqatCvAUfKttLearuWrP9MDH5MBPbIqV92AaeXatLxBI9gBaebbnrfifHhDYfgasaacH8akY=wiFfYdH8Gipec8Eeeu0xXdbba9frFj0=OqFfea0dXdd9vqai=hGuQ8kuc9pgc9s8qqaq=dirpe0xb9q8qiLsFr0=vr0=vr0dc8meaabaqaciaacaGaaeqabaqabeGadaaakeaacuqGZbWCgaacamaaBaaaleaacqqGNbWzaeqaaaaa@2FAA@ is the modified gene variance and *ν*_*g *_is the inverse of the number of degrees of freedom for gene g. A T-score threshold is chosen, and the FDR is the ratio between the number of genes above the cut-off on the self-self list and high contrast list. To find genes at a specific false discovery rate (e.g. 0.05), T-scores computed for the high contrast and the self-self experiment are sorted and the T-score cut-off is lowered until the chosen false discovery rate is obtained (Figure 1). The number of genes found at a standard false discovery rate (e.g. 5 %) may then be used as an optimization criterion to be maximized. It is important to note that this method of determining FDR rates relies on a large number of truly differentially expressed genes in the high contrast experiment and should not be used as a tool for analysis of biological experiments where only a few genes may be differentially expressed.

### Applications

There is a large number of steps in the microarray production, hybridization, scanning and data analysis that have adjustable parameters that may be optimized. We will show how HCSSM can be used to optimize data analysis and hybridization protocols. As mentioned above, the standard microarray data analysis pipeline consists of image analysis, filtering of data, background correction, normalization and identification of differentially expressed genes. Recent studies have focused on the normalization step and evaluated a large number of normalization methods for cDNA microarray data [15,16]. We have therefore chosen to test the HCSSM on filtering and background correction, as these are fields where there is still considerable debate in the literature [4,17].

#### Effects of level of filtration on the number of differentially expressed genes

It is common in microarray analysis to reduce the impact of spots that are malformed or have intensities outside the linear range of the scanner. Such spots are either removed, or weighted down using some measure of spot quality. Filtering has been shown to introduce bias in microarray studies [18], but has also been shown to significantly reduce variation in self-self hybridizations [13]. However, as far as we know, no work has shown how the *bias versus variance *trade-off in practice influences the ability to detect differentially expressed genes. Therefore, we tested a number of different filtering methods of increasing complexity to evaluate how they influence the ability to find differentially expressed genes in a study.

Often filtering consists of removing spots with intensities below a (arbitrary) threshold [19]. We have however found no investigations into the effect of such filtering. Several filtering methods based on spot quality statistics have been reported [13,18,20]. These quality measures can be used as filters by setting a spot quality threshold, but can also be used as weights to reduce the impact of low quality spots.

We propose a simple quality measure using the variability information available from most image analysis software. The variation in a log ratio (*S*_*M*_) can be approximated from the variation in the pixel intensities using Equation (2) [19]

SM=1G×ln⁡(2)σg2+1R×ln⁡(2)σR2
 MathType@MTEF@5@5@+=feaafiart1ev1aaatCvAUfKttLearuWrP9MDH5MBPbIqV92AaeXatLxBI9gBaebbnrfifHhDYfgasaacH8akY=wiFfYdH8Gipec8Eeeu0xXdbba9frFj0=OqFfea0dXdd9vqai=hGuQ8kuc9pgc9s8qqaq=dirpe0xb9q8qiLsFr0=vr0=vr0dc8meaabaqaciaacaGaaeqabaqabeGadaaakeaacqWGtbWudaWgaaWcbaGaemyta0eabeaakiabg2da9maakaaabaWaaSaaaeaacqaIXaqmaeaacqWGhbWrcqGHxdaTcyGGSbaBcqGGUbGBcqGGOaakcqaIYaGmcqGGPaqkaaacciGae83Wdm3aa0baaSqaaiabdEgaNbqaaiabikdaYaaakiabgUcaRmaalaaabaGaeGymaedabaGaemOuaiLaey41aqRagiiBaWMaeiOBa4MaeiikaGIaeGOmaiJaeiykaKcaaiab=n8aZnaaDaaaleaacqWGsbGuaeaacqaIYaGmaaaabeaaaaa@4CC6@

Where *G *and *R *are the green and red signal intensities, *σ*_*R*_, *σ*_*G *_are the pixel standard deviations in the red and green channel respectively.

Quality weighting can be obtained by:

w=1(SM+∂)
 MathType@MTEF@5@5@+=feaafiart1ev1aaatCvAUfKttLearuWrP9MDH5MBPbIqV92AaeXatLxBI9gBaebbnrfifHhDYfgasaacH8akY=wiFfYdH8Gipec8Eeeu0xXdbba9frFj0=OqFfea0dXdd9vqai=hGuQ8kuc9pgc9s8qqaq=dirpe0xb9q8qiLsFr0=vr0=vr0dc8meaabaqaciaacaGaaeqabaqabeGadaaakeaacqWG3bWDcqGH9aqpdaWcaaqaaiabigdaXaqaaiabcIcaOiabdofatnaaBaaaleaacqWGnbqtaeqaaOGaey4kaSIaeyOaIyRaeiykaKcaaaaa@36AB@

The delta term is a small number added to stabilize spots with very small uncertainties.

#### Effects of background correction on the number of differentially expressed genes

The rationale of background correction is that the e.g. observed green signal intensity (*G*) is a linear combination of a true signal coming from the labelled RNA (*G*_*t*_) and a background signal (*G*_*b*_) so that:

*G *= *G*_*t *_+ *G *_*b*_

More accurate log ratios can then be obtained by correcting the observed signal by subtracting the background. The problem then is to correctly estimate the background signal for each spot in the image analysis phase, and correcting this estimate to satisfy prior criteria e.g. background smoothness and non-negativity of the true signal. Background correction in itself may also destabilize results by increasing variance [5], and may decrease the ability to detect differentially expressed genes. Therefore, we use the HCSSM to compare the ability to find differentially expressed genes when no background correction is used *versus *a non negative background correction method [21].

#### Effect of choice and dose of dT blockers in cDNA microarray experiments

The HCSS-method can be applied to the laboratory procedures as well as to the data processing steps in microarray research. One major source of bias in cDNA microarrays is cross-hybridization of the labelled RNA to non-target homologous probe sequences on the array [22-24]. A substantial proportion of the non-specific cross-hybridization signal is due to poly(dA)-poly(dT) cross hybridization since poly(dT)-containing molecules produced during labelling of poly(dA) tails of sample RNAs by reverse transcription can bind promiscuously to the poly(dA) stretch of cDNA probe spots. These poly(dA) hybridization signals reduce the ability to detect differentially expressed genes. Thus, minimization of poly(dA) hybridization is a major challenge in cDNA microarray experiments. To reduce the poly(dA) signals it is common practice to add a blocker like synthetic poly(dA) in the probe or poly(dT) in the hybridization mixture [25-27]. Recently, an LNA dT Blocker containing Locked Nucleic Acid (LNA) nucleotides [28] has been introduced. There is currently no standardized protocol available that can be used to evaluate and optimize blocking procedures with respect to optimizing the observed number of differentially expressed genes. In the present study we therefore compared the effect of poly(dA)_40–60 _and different doses of LNA dT blocker on the number of differentially expressed genes, and used the HCSSM to optimize identification of truly differentially expressed genes.

## Results and discussion

### The choice and dose of blocking reagent influences the number of differentially expressed genes estimated

In order to evaluate how different conditions designed to block poly(dA)-poly(dT) cross hybridization affect the ability to detect differentially expressed genes, we applied the HCSS-method to analysis of microarray experiments performed in the presence of either poly(dA)_40–60 _or varying amounts of LNA. Poly(dA)_40–60 _binds to the poly(dT) segment of the labelled target in the hybridization solution and thus competes with the poly(dA) segment in the microarray cDNA probes. LNA dT blocker contains Locked Nucleic Acid (LNA) nucleotides [28] at key positions within the (dT) synthetic strand, and is designed to block poly (dA) sequences present in the microarray cDNA probes and prevent them from hybridizing to poly(dT) segment of the labelled targets.

**Figure 2:**
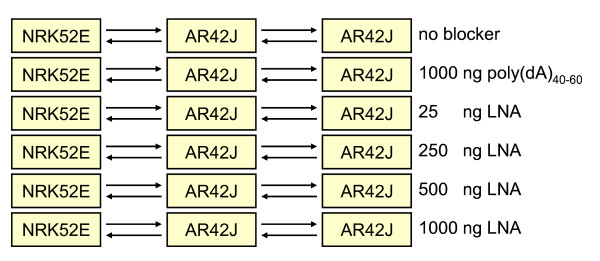
**Graphical representation of experimental design of the microarray experiment**. The nodes correspond to RNA from samples with high expected differential gene expression (rat cell lines NRK52E and AR42J) compared to self-self hybridization (rat cell line AR42J). The samples were hybridized to rat 15 k cDNA duplicates under six different blocking conditions including no blocker, 1000 ng poly(dA)_40–60_, and 25 to 1000 ng LNA dT blocker. Dye-swap and self *versus *self were performed for all blocking conditions (total of 24 hybridizations). Green-labelled samples are placed at the tail and red labelled samples at the head of the arrows.

**Figure 3:**
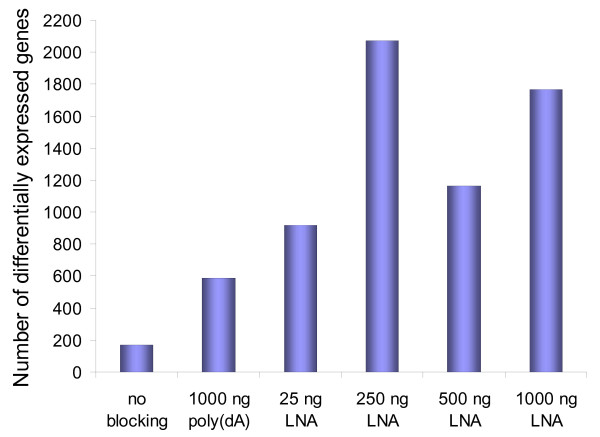
**Effect of choice and dose of dT blocker on the number of differentially expressed genes**. The figure shows data analysed with *weighted filtration *and *no background correction *(see Methods section).

Six different blocking conditions with dye-swap and self *versus *self were investigated in a total of 24 hybridizations (Figure 2). Significantly differentially expressed genes were determined by estimating the false discovery rate using a null distribution obtained from the self-self experiments, as described in the Background section. Filtration was performed as described below and in the Methods section. Our results clearly demonstrate the effect of blocking the poly(dT) segment of the labelled target since addition of poly(dA)_40–60 _increased the number of estimated differentially expressed genes from 167 (no poly dA/dT blocking reagent) to 588. The LNA dT blocker further increased the number of estimated differentially expressed genes in a dose dependent manner with a maximum of 2064 differentially expressed genes estimated in the presence of 250 ng LNA (Figure 3). In general, more than five biological experiments are recommended for detecting differentially expressed genes in microarry experiments [4]. Technical replicates are mainly recommended in quality-control studies. We would like to emphasize that even our HCSS-method only requires four microarrays per evaluation; more experiments are needed to accurately determine whether the spike in differentially expressed genes for the 250 ng LNA polyA is a real optimum or noise in the dose response curve. It is also important to notice that the optimal dose and choice of blocker may vary for different arrays and microarray protocols. However, our results indicate that the concept of using self-self and high contrast hybridizations in control experiments is well suited to identify optimal blocking conditions.

### Background correction method has a stronger impact on the estimated number of differentially expressed genes than filtering

**Figure 4:**
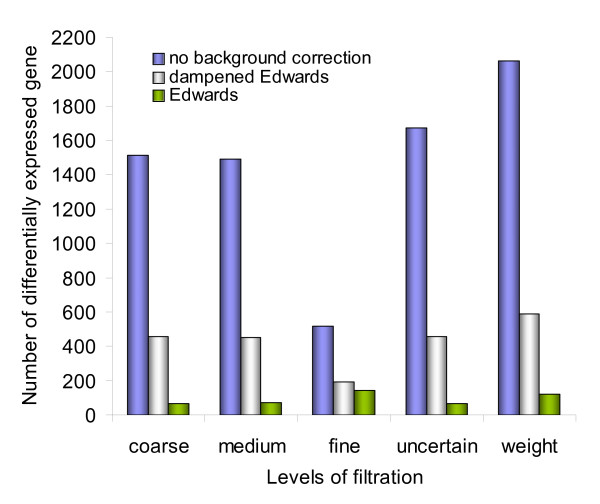
**Effects of background correction and level of filtration on the number of differentially expressed genes estimated**. The figure shows data from hybridization with 250 ng LNA blocker added.

The data from the blocking study were also analysed using combinations of filter methods and background correction (Figure 4 and Additional file 1). We tested the following five different filtering methods of increasing complexity: *coarse*, *medium*, *fine*, *uncertain *and *weighting filter*. Filters were based on spot foreground intensity, percentage of pixels saturated and ratio uncertainties as described in the Methods section. In addition we applied three different background corrections: *none *were only foreground signal is used to calculate ratios, *Edwards *which is a subtraction method that ensures positive values [21] and *dampened Edwards *were a small number is added to the corrected signal to avoid extreme ratios in those cases where the background corrected intensities would be very low. *Weight based filtering*; using the ratio uncertainty to reduce the impact of "bad" spots without removing them completely found the highest numbers of differentially expressed genes regardless of background correction method and blocking agent dose (Figure 4 and Addition file 1). The choice of background correction method had a higher impact on the number of differentially expressed genes than filtering. *No background *correction resulted in the highest amount of differentially expressed genes estimated, the numbers ranging from 515 to 2064 under the five different filtering methods and blocking with 250 ng LNA (Figure 4). *Edwards *and *dampened Edwards *gave 66–120 and 191–588 differentially expressed genes, respectively, under the same filtering conditions. Our results strongly indicate that omission of background correction consistently improves the results. The reason may be that background correction introduces a lot of variability to remove a small bias. As long as the red and green backgrounds are highly correlated they will dampen the ratios, but only significantly for the low intensity spots, for which the ratios are uncertain anyway. One strategy may be to use error propagation to evaluate background correction, and only background correct the spots where the bias is large, thus not increasing the variance of all spots, but still removing the bias for some of the worst affected spots.

### Validation of cDNA microarray data generated by HCSSM

**Figure 5:**
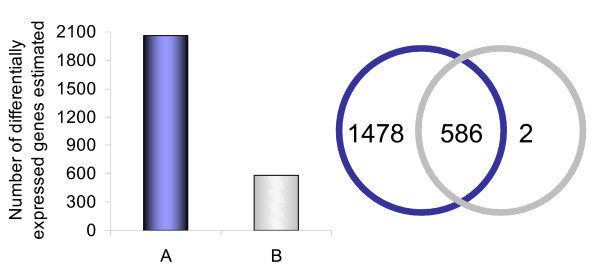
**Comparison of gene lists generated with different background correction methods**. The left figure illustrates the number of differentially expressed genes estimated with *no background correction *(A) and *dampened Edwards background correction *(B). The Venn diagram shows overlap of differentially expressed genes estimated with *no background correction *(blue) and *dampened Edwards *(grey). All data are from hybridization with 250 ng LNA blocker added and *weight based filtering*.

In our experiments a dose of 250 ng LNA dT blocker gave the highest number of differentially expressed genes. When HCSSM was further used to evaluate steps in the computational analysis of microarray data we found that *no background correction *and *weight based filtering *estimated 2064 differentially expressed genes (gene list A in Figure 5) compared to 588 when the data was analysed by *dampened Edwards background correction *and *weight based filtering *(gene list B in Figure 5). Thus, omission of background correction added ~1478 unique genes to the list of genes identified as differentially expressed. Only 2 of the 588 genes identified with *dampened Edwards background correction *and *weight based filtering *were not detected when *no background correction *was performed (Figure 5).

In order to investigate the level of concordance of biological themes represented in the gene lists A and B, we used GeneTools, an "all in one" annotation web tool package [29] recently described by Beisvag et al, 2006 [30]. The two gene lists (reporter lists) were submitted to *e*GOn (explore genontology), which automatically associates Gene Ontology (GO) terms from public databases to the submitted gene list. We found that the main GO terms were represented in both gene list A and gene list B. Furthermore, the proportion (number of genes in A/number of genes in B) associated with specific GO terms was similar for all terms, as illustrated for selected GO terms in Table 1. The average proportion for all GO terms associated with five or more genes was 2.9 ± 0.8, n = 64 (Additional file 2). This indicated that the increase in differently expressed genes estimated by HCSSM was not random. We than applied cross platform analysis to validate that the 1478 genes added to the gene list was real.

**Table 1:** Selected GO categories associated with genes differentially expressed in the cell lines AR42J *versus *NRK52E.

**GO number**	**GO term**	**A**	**B**	**AUB**	**BÚA**	**AÚB**	**Proportion**
GO:0008150	biological process	724	233	233	491	0	**3.1**
GO:0008152	metabolic process	459	135	135	324	0	**3.4**
GO:0032502	developmental process	246	91	91	155	0	**2.7**
GO:0007154	cell communication	202	69	69	133	0	**2.9**
GO:0050896	response to stimulus	165	64	64	101	0	**2.6**
GO:0048468	cell development	112	38	38	74	0	**2.9**
GO:0006950	response to stress	101	32	32	69	0	**3.2**
GO:0009056	catabolic process	65	30	30	35	0	**2.2**
GO:0007049	cell cycle	60	13	13	47	0	**4.6**
GO:0016265	death	60	18	18	42	0	**3.3**
GO:0006928	cell motility	51	17	17	34	0	**3.0**
GO:0040007	growth	42	19	19	23	0	**2.2**
GO:0006952	defence response	32	7	7	25	0	**4.6**
GO:0019725	cell homeostasis	30	12	12	18	0	**2.5**

**A: **Gene list generated with *no background correction *and *weight based filtering*. **B: **Gene list generated with *dampened Edwards background correction *and *weight based filtering*. **AUB: **number of genes differentially expressed in both gene list A and B. **AÚB: **number of genes differentially expressed in gene list A but not in gene list B. **BÚA: **number of genes differentially expressed in gene list B but not in gene list A. **Proportion**: number of genes in A/number of genes in B. The genes were annotated using GeneTools [30] according to UniGen build #163. All GO categories associated with more than five genes are shown in Additional file 2.

#### Cross platform microarray analysis

**Figure 6:**
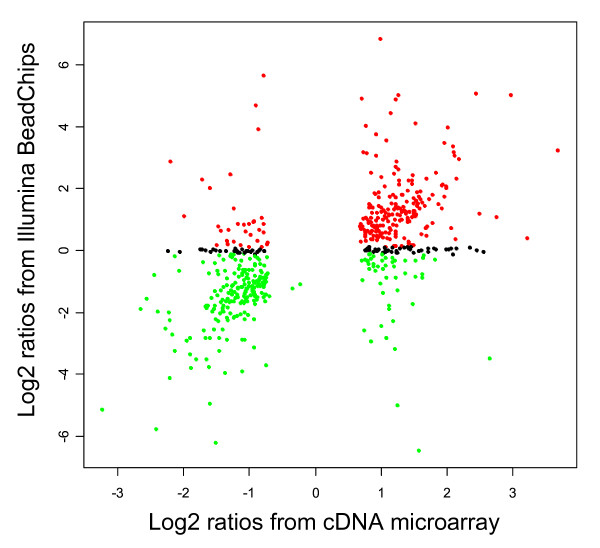
**Cross platform microarray analysis**. The figure shows ratios (log2 based) from 553 genes comparable between the cDNA and the Illumina platforms. Red dots: genes significantly up-regulated on the Illumina platform. Green dots: genes significantly down-regulated on the Illumina platform. Black dots: genes not identified as significantly different between the cell lines (AR42J *versus *NRK52E) on the Illumina platform. All the genes were identified as differentially expressed on the cDNA platform, and the concordance between cDNA and Illumia is 72 %.

The combined use of multiple microarray platforms has recently been suggested as an alternative that is complementary to qRT-PCR for validation of gene expression profiles [31-33]. In an attempt to validate a large fraction of the cDNA microarray results we used the Illumina Gene Expression system (San Diego, CA) for cross-platform analysis of differentially expressed genes in AR42J cells *versus *NRK52E cells. The same sources of total RNA used in the cDNA microarray experiments were hybridized to Illumina's Sentrix^® ^RatRef-12 Expression BeadChips, with six technical replicates for each cell line as described in the Methods section. Only genes common to both platforms were included in the analysis. Of the 1478 additional genes identified with *no background correction*, 553 genes were represented on both the spotted cDNA arrays and the Illumina RatRef-12 Expression BeadChips. Of these 553 genes, 398 (72 %) were identified as differentially expressed in the same direction (higher *versus *lower) on both platforms (Fig 6). The overlaps of differentially expressed genes across commercially microarray platforms are recently reported to be~80–90 % [10,32,33].

Since RT-PCR is still considered the "golden standard" for gene expression measurements, we further used SYBR green-based quantitative real-time PCR to validate the relative gene expression for a few of the 1478 additional genes identified with optimal conditions. Commonly, a 2 fold change is reported as the cut-off below which microarray and qRT-PCR data begin to loose correlation, although recent reports observed significant correlation where genes exhibited 1.4 fold change or higher [34,35]. We therefore selected seven genes in the fold ratio range 4.8 to 1.8 (log2 ratio range 2.2 to 0.9 on the cDNA arrays). Results are shown in Additional files 3 and 4. Description of PCR primers and qRT-PCR protocol are shown in Additional file 5. Five of the selected genes (*Ica1, c-fos, Btg2, Uhrf and Ube2b*) were detected as differentially expressed on both microarray platforms. Two genes (*Hoxa2b *and *Irfrd*) were only identified on the the cDNA platform. Five genes with fold ratio > 2 were verified by qRT-PCR (see Additional files 3 and 4). Two genes (*Hoxa2 *and *Ube2b*) with fold ratio < 2 were not verified. *Hox2b *was excluded from the results due to technical problems (see Additional file 4). The gene *Ube2b *was expressed in the opposite direction relative to the cDNA microarray, but in the same direction as on the Illumina platform (higher in AR42J than in NRK52E on Illumina, lower on cDNA). Consistent with other reports [35,36], the microarray ratios were compressed compared to qRT-PCR results (see Additional file 3). Overall, the qRT-PCR measurement corresponded 100 % (5/5) with the Illumina data and 71 % (5/7) with the cDNA microarray data. The latter confirmation rate is in accordance with cDNA microarray results of others ([37], and references therein).

Some of the discrepancy between cDNA microarry *versus *Illumina and qRT-PCR may be due to errors (reported by e.g. [38]) in the IMAGE collection used for probes in our cDNA microarrays. We thus consider the results from qRT-PCR and the Illumina platform more likely to be "true data" in the present study. In spite of the known errors on cDNA platforms, cross platform analysis confirmed 72 % of the results. Validation of a few selected genes by qRT-PCR supported this finding. Taken together, functional annotation analysis and the alternative methods for validation of the cDNA micoarry data indicated that the increase in the number of differentially expressed genes found by HCSSM was mainly real.

## Conclusion

In the present study we have used the HCSSM in cDNA microarray control experiments to evaluate blocking conditions, filtration and background correction. We found that the choice and dose of poly(dT)-poly(dA) blocking agents during hybridization influenced the estimated number of differentially expressed genes. When HCSSM was used to evaluate steps in the computational analysis of microarray data we found that *no background correction *and *weight based filtering *gave the largest estimated numbers of differentially expressed genes. The background correction method, however, has a stronger impact on the numbers of differentially expressed genes found than filtering. The results show that HCSSM may be a useful and simple approach to optimize cDNA microarrays procedures without including external standards.

## Methods

### Cells

AR42J (rat pancreatic acinar cell derived, ATCC, Rockville, MD) were grown in Dulbecco's modified Eagle's medium (DMEM) with 4.5 g/l glucose (Invitrogen, Carlsbad, CA), 15 % fetal calf serum (Euroclone Ltd, Devon, UK), 1 mM sodium pyruvate, 0.1 mg/ml L-glutamine (Invitrogen, Carlsbad, CA), 10 U/ml penicillin/streptomycin (Invitrogen, Carlsbad, CA), and 1 *μ*g/ml fungizone (Invitrogen Carlsbad, CA). NRK52E cells (rat kidney epithelial cells, ATCC, Rockville, MD) were grown in DMEM with 4.5 g/l glucose (Invitrogen, Carlsbad, CA) supplemented with 5 % FCS (Euroclone Ltd), 0.1 mg/ml L-glutamine (Invitrogen, Carlsbad, CA) and 10 U/ml penicillin/streptomycin (Invitrogen, Carlsbad, CA). All cells were grown at 37°C in a humidified atmosphere at 5 % CO_2_.

### Treatment of cells and isolation of RNA

AR42J and NRK52E cells were cultured in 75 cm^2 ^culture flasks for 72 h until confluence was reached. Total RNA was isolated using RNeasy^® ^Midi kit (Qiagen, Germantown, MD) according to the manufacturer's instruction. RNA was quantified and assessed for purity by measurement of OD260 and OD280 using a UV fiberoptic spectrophotometer (Nanodrop Technologies, Rockland, DE) and was qualitatively assessed by measurement of relative 28S and 18S ribosomal band intensities using a Bioanalyzer and RNA LabChip capillary gel electrophoresis assay (Agilent Technologies, Palo Alto, CA). Aliquots were kept frozen at -80°C until further processing.

### cDNA Microarray procedure

cDNA Microarrays were manufactured by the Norwegian Microarray Consortium [39] using 15000 cDNA rat probes from Research Genetics (IMAGE collection) printed in duplicates on Corning CMT Gaps II slides (Corning Inc. NY). The probes were dissolved in 50 % DMSO to ensure high printing quality. The microarray slides were UV cross linked at 300 mJ in order to fix the DNA to the glass slides.

Labelling was performed by reverse transcription of total RNA from the cell lines (3 *μ*g each) in the presence of primers containing the capture sequence for subsequent hybridization of Cy3- and Cy5-labelled dendrimers, using the Genisphere 3DNA Array 350 Expression Array Detection kit (Genisphere, Hatfield, PA) as described in the manufacturer's protocol. Different combinations of blocking reagents were added to the hybridization mixture: Mouse COT-1 DNA (Life Technologies) was added to all mixtures (0.1 *μ*g/*μ*g RNA) in order to block hybridization of repetitive elements. Different concentrations (0.1–4.0 *μ*l = 25–1000 ng) of LNA dT Blocker (Genisphere, Hatfield, PA) and 1 *μ*l = 1000 ng Poly(dA)_40–60 _(Stratagen Spotreporter; La Jolla, CA) were used as illustrated in Figure 2.

Hybridization was done in a humidified hybridization chamber (Corning Inc., NY) at 60°C for 14–15 h in a total hybridization volume of 60 *μ*l. Post hybridization washes were done for 15 min at 55°C with 2× SSC and 0.2 % SDS, for 10 min at room temperature with 2× SSC, and finally for 10 min at room temperature with 0.2× SSC. After the washing, the Cy3- and Cy5-labelled dendrimers were hybridized to the capture sequence at the reverse transcribed sample RNA at 60°C for 3 h as described in the manufacturer's protocol. After dendrimer hybridization, the slides were washed as described above and dried by centrifugation at 1500 rpm for 5 min.

### Scanning and image analysis of cDNA microarrays

The slides were scanned at a resolution of 10 *μ*m by use of Packard Bioscience Scanarray Express HT scanner (Packard BioScience, Billerica, MA). A laser power of 100 % was used, and excitation of Cy3 and Cy5 was performed at a wavelength of 532 nm and 635 nm, respectively. Signals were detected by use of photomultiplier tubes (PMTs) with two channels. In order to maximizing the certainty of the weakest spots, the PMT voltage was adjusted to keep the background intensity between 200 and 400 (mean spot intensity) in each channel as suggested in [40]. The GenePix 5.0 image analysis software (Axon Instruments, Inc., Union City, CA) was used for spot segmentation and intensity calculations. Spots and regions with high unspecific binding of dye or dust particles were manually flagged and excluded from the analysis.

### Data analysis of cDNA microarrays

Modified T-scores and false discovery rate (FDR) values were calculated as described under Applications in the Background section for all combinations of filter, background corrections, and blocking treatments.

#### Filtering methods

A progression of increasingly complex filter functions was evaluated.

*Coarse filter*: Spots flagged as bad or missing during the image analysis are removed.

*Medium filter*: Also removes spots with intensities below 200 and spots with more than 70 % saturation in both channels.

*Fine filter*: Also removes spots that are small, i.e. spots with diameters less than 20.

*Uncertain filter*: Removes spots flagged by GenePix 5.0 as "bad" or "missing" and spots with deltaM > 3.

*Weight based filter***: **Quality weighting can be obtained by using Equation (3) as described in the Background section:

w=1(SM+∂)(delta=0.5)
 MathType@MTEF@5@5@+=feaafiart1ev1aaatCvAUfKttLearuWrP9MDH5MBPbIqV92AaeXatLxBI9gBaebbnrfifHhDYfgasaacH8akY=wiFfYdH8Gipec8Eeeu0xXdbba9frFj0=OqFfea0dXdd9vqai=hGuQ8kuc9pgc9s8qqaq=dirpe0xb9q8qiLsFr0=vr0=vr0dc8meaabaqaciaacaGaaeqabaqabeGadaaakeaafaqabeqacaaabaGaem4DaCNaeyypa0ZaaSaaaeaacqaIXaqmaeaacqGGOaakcqWGtbWudaWgaaWcbaGaemyta0eabeaakiabgUcaRiabgkGi2kabcMcaPaaaaeaacqGGOaakcqqGKbazcqqGLbqzcqqGSbaBcqqG0baDcqqGHbqycqGH9aqpcqaIWaamcqGGUaGlcqaI1aqncqGGPaqkaaaaaa@42F1@

The delta term is a small number added to stabilize spots with very small uncertainties.

### Background correction methods

Three different background corrections strategies were applied:*None *were only foreground signal is used to calculate ratios, *Edwards *which is a subtraction method that ensures positive values [21] and *dampened Edwards *were a small number (e.g. 20) is added to the corrected signal to avoid extreme ratios in those cases where the background corrected intensity would be very low.

### Statistical analysis

After filtering and background correction, all arrays were normalized using loess to remove intensity dependent variations in the ratios [41]. Differentially expressed genes were found at the 5 % FDR level using the methodology described in the Background section.

#### Cross-platform microarray analysis

The Illumina Gene Expression system was used for cross-platform analysis of differentially expressed genes in AR42J cells *versus *NRK52E cells. RNA amplifications and hybridization were performed at the Finnish DNA Microarray Centre at Turku Centre of Biotechnology [42]. Briefly, aliquots from the same RNA as in the cDNA microarray experiments were amplified with Ambion's Illumina^® ^TotalPrep RNA Amplification kit (cat no AMIL1791) using 400 ng of total RNA as input material. The in vitro transcription (IVT) amplification that incorporated biotin-labelled nucleotides was performed overnight (14 h) at 37°C. After the amplifications the cRNA concentrations where checked with NanoDrop ND-1000 and cRNA quality was controlled by BioRad's Experion electrophoresis station.

A total of 750 ng of each biotin-labelled cRNA sample was hybridized to Illumina's Sentrix^® ^RaRef-12-v1 Expression BeadChips at 58°C overnight (17 h) according to the manufacturer's instructions. The hybridized biotinylated cRNA was detected with 1 *μ*g/ml Cyanine3-streptavidine, (GE Healthcare Biosciences; cat no PA43001) and the Beadchips were scanned with Illumina BeadArray Reader (Factor = 1, PMT = 521, Filter = 100 %). Numerical results were extracted with Bead Studio v3.0.19.0 without any normalization or background subtraction.

The Illumina data were analysed using a modified T-test [14] with p-values adjusted for false discovery rate [43], and genes with adjusted p-values < 0.05 were taken as significant. Entrez Gene IDs were used as common identifiers for the microarray platforms. The genes were identified according to UniGen build #163 in GeneTools [29,30].

### Database submission of microarray data

The microarray data were prepared according to minimum information about a microarray experiment (MIAME) recommendations [44] and deposited in the Array Express [45]. Detailed information about the microarray designs (platforms) and raw data files from the experiments are accessible in ArrayExpress by use of these accession numbers; **A-MEXP-358 **(cDNA platform), **E-TABM-202 **(cDNA experiment) and **E-TABM-307 **(Illumina experiment).

## Authors' contributions

TB and EA contributed equally to this work, conceived the study and wrote the manuscript. BDE, HB and VB contributed with ideas on the microarray experiments and revised the manuscript. BDE and TB performed the cDNA microarray experiments and cDNA image analysis. TB performed and analysed the qRT-PCR assays. EA performed the data analysis and tested and improved the statistics. HB produced the spotted cDNA microarrays and performed the database submission of the cDNA microarray data. VB investigate the level of concordance of biological themes represented in different gene lists, and performed the database submission of the data from the Illumina Gene Expression system. AL supervised the study and revised the manuscript. All authors have read and approved the final manuscript.

## Supplementary Material

Additional file 1 Table S1. Number of differentially expressed genes under different blocking, background correction and levels of filtration. The file shows all combinations of blocking, background correction and levels of filtration together with the estimated numbers of differentially expressed genes with the different combinations. Click here for file

Additional file 2 Table S2. Level of concordance of biological themes represented in the gene list generated with different background correction methods. The file shows all GO terms at hierarchy level 1–3 which was associated with five or more genes(n = 64). Click here for file

Additional file 3 Figure S1. Validation of differentially expressed genes by SYBR green-based quantitative real-time PCR. The figure shows fold changes for a few selected genes on the microarry platforms and the correspondence with relative gene expression data from qRT-PCR assays. Click here for file

Additional file 4 Cross platform microarray analysis (Table S3a) and SYBR green-based quantitative real-time PCR (Table S3b). The file shows log2 ratio differences and fold ratios for a few selected genes on the microarray platforms (Table S3a) compared to ΔΔCt values and calculated fold change values from validation by qRT-PCR (Table S3b). Click here for file

Additional file 5 PCR primers (Table S4) and qRT-PCR protocol. The file gives information about the PCR primers and the qRT-PCR protocol used in the study. Click here for file

## References

[B1] Sandvik AK, Alsberg BK, Norsett KG, Yadetie F, Waldum HL, Laegreid A (2006). Gene expression analysis and clinical diagnosis. Clin Chim Acta.

[B2] Ge H, Walhout AJM, Vidal M (2003). Integrating `omic' information: a bridge between genomics and systems biology. Trends in Genetics.

[B3] Kitano H (2002). Systems biology: a brief overview. Science.

[B4] Allison DB, Cui X, Page GP, Sabripour M (2006). Microarray data analysis: from disarray to consolidation and consensus. Nat Rev Genet.

[B5] Andersen MT, Foy CA (2005). The development of microarray standards. Anal Bioanal Chem.

[B6] Novoradovskaya N, Whitfield ML, Basehore LS, Novoradovsky A, Pesich R, Usary J, Karaca M, Wong WK, Aprelikova O, Fero M, Perou CM, Botstein D, Braman J (2004). Universal Reference RNA as a standard for microarray experiments. BMC Genomics.

[B7] van Bakel H, Holstege FC (2004). In control: systematic assessment of microarray performance. EMBO Rep.

[B8] Kawasaki ES (2006). The End of the Microarray Tower of Babel: Will Universal Standards Lead the Way?. J Biomol Tech.

[B9] Brazma A, Krestyaninova M, Sarkans U (2006). Standards for systems biology. Nat Rev Genet.

[B10] Shi L, Reid LH, Jones WD, Shippy R, Warrington JA, Baker SC, Collins PJ, de Longueville F, Kawasaki ES, Lee KY, Luo Y, Sun YA, Willey JC, Setterquist RA, Fischer GM, Tong W, Dragan YP, Dix DJ, Frueh FW, Goodsaid FM, Herman D, Jensen RV, Johnson CD, Lobenhofer EK, Puri RK, Schrf U, Thierry-Mieg J, Wang C, Wilson M, Wolber PK, Zhang L, Amur S, Bao W, Barbacioru CC, Lucas AB, Bertholet V, Boysen C, Bromley B, Brown D, Brunner A, Canales R, Cao XM, Cebula TA, Chen JJ, Cheng J, Chu TM, Chudin E, Corson J, Corton JC, Croner LJ, Davies C, Davison TS, Delenstarr G, Deng X, Dorris D, Eklund AC, Fan XH, Fang H, Fulmer-Smentek S, Fuscoe JC, Gallagher K, Ge W, Guo L, Guo X, Hager J, Haje PK, Han J, Han T, Harbottle HC, Harris SC, Hatchwell E, Hauser CA, Hester S, Hong H, Hurban P, Jackson SA, Ji H, Knight CR, Kuo WP, LeClerc JE, Levy S, Li QZ, Liu C, Liu Y, Lombardi MJ, Ma Y, Magnuson SR, Maqsodi B, McDaniel T, Mei N, Myklebost O, Ning B, Novoradovskaya N, Orr MS, Osborn TW, Papallo A, Patterson TA, Perkins RG, Peters EH, Peterson R, Philips KL, Pine PS, Pusztai L, Qian F, Ren H, Rosen M, Rosenzweig BA, Samaha RR, Schena M, Schroth GP, Shchegrova S, Smith DD, Staedtler F, Su Z, Sun H, Szallasi Z, Tezak Z, Thierry-Mieg D, Thompson KL, Tikhonova I, Turpaz Y, Vallanat B, Van C, Walker SJ, Wang SJ, Wang Y, Wolfinger R, Wong A, Wu J, Xiao C, Xie Q, Xu J, Yang W, Zhang L, Zhong S, Zong Y, Slikker W (2006). The MicroArray Quality Control (MAQC) project shows inter- and intraplatform reproducibility of gene expression measurements. Nat Biotechnol.

[B11] Han T, Melvin CD, Shi L, Branham WS, Moland CL, Pine PS, Thompson KL, Fuscoe JC (2006). Improvement in the Reproducibility and Accuracy of DNA Microarray Quantification by Optimizing Hybridization Conditions. BMC Bioinformatics.

[B12] Thompson KL, Rosenzweig BA, Pine PS, Retief J, Turpaz Y, Afshari CA, Hamadeh HK, Damore MA, Boedigheimer M, Blomme E, Ciurlionis R, Waring JF, Fuscoe JC, Paules R, Tucker CJ, Fare T, Coffey EM, He Y, Collins PJ, Jarnagin K, Fujimoto S, Ganter B, Kiser G, Kaysser-Kranich T, Sina J, Sistare FD (2005). Use of a mixed tissue RNA design for performance assessments on multiple microarray formats. Nucleic Acids Res.

[B13] Hautaniemi S, Edgren H, Vesanen P, Wolf M, Jarvinen AK, Yli-Harja O, Astola J, Kallioniemi O, Monni O (2003). A novel strategy for microarray quality control using Bayesian networks. Bioinformatics.

[B14] Smyth GK (2004). Linear models and empirical bayes methods for assessing differential expression in microarray experiments. Stat Appl Genet Mol Biol.

[B15] Wu W, Xing EP, Myers C, Mian IS, Bissell MJ (2005). Evaluation of normalization methods for cDNA microarray data by k-NN classification. BMC Bioinformatics.

[B16] Fujita A, Sato J, Rodrigues L, Ferreira C, Sogayar M (2006). Evaluating different methods of microarray data normalization. BMC Bioinformatics.

[B17] Qin LX, Kerr KF (2004). Empirical evaluation of data transformations and ranking statistics for microarray analysis. Nucleic Acids Res.

[B18] Tom BD, Gilks WR, Brooke-Powell ET, Ajioka JW (2005). Quality determination and the repair of poor quality spots in array experiments. BMC Bioinformatics.

[B19] Quackenbush J (2002). Microarray data normalization and transformation. Nat Genet.

[B20] Bylesjo M, Eriksson D, Sjodin A, Sjostrom M, Jansson S, Antti H, Trygg J (2005). MASQOT: a method for cDNA microarray spot quality control. BMC Bioinformatics.

[B21] Edwards D (2003). Non-linear normalization and background correction in one-channel cDNA microarray studies. Bioinformatics.

[B22] Flikka K, Yadetie F, Laegreid A, Jonassen I (2004). XHM: a system for detection of potential cross hybridizations in DNA microarrays. BMC Bioinformatics.

[B23] Chuaqui RF, Bonner RF, Best CJ, Gillespie JW, Flaig MJ, Hewitt SM, Phillips JL, Krizman DB, Tangrea MA, Ahram M, Linehan WM, Knezevic V, Emmert-Buck MR (2002). Post-analysis follow-up and validation of microarray experiments. Nat Genet.

[B24] Chen Y, Chou CC, Lu X, Slate E, Peck K, Xu W, Voit E, Almeida J (2006). A multivariate prediction model for microarray cross-hybridization. BMC Bioinformatics.

[B25] Handley D, Serban N, Peters D, O'Doherty R, Field M, Wasserman L, Spirtes P, Scheines R, Glymour C (2004). Evidence of systematic expressed sequence tag IMAGE clone cross-hybridization on cDNA microarrays. Genomics.

[B26] Nguyen C, Rocha D, Granjeaud S, Baldit M, Bernard K, Naquet P, Jordan BR (1995). Differential gene expression in the murine thymus assayed by quantitative hybridization of arrayed cDNA clones. Genomics.

[B27] Pan SJ, Rigney DR, Ivy JL (2002). Outliers involving the Poly(A) effect among highly-expressed genes in microarrays. BMC Genomics.

[B28] Vester B, Wengel J (2004). LNA (locked nucleic acid): high-affinity targeting of complementary RNA and DNA. Biochemistry.

[B29] GeneTools - a collection of resources for functional annotations. http://www.genetools.microarray.ntnu.no/common/intro.php.

[B30] Beisvag V, Junge FK, Bergum H, Jolsum L, Lydersen S, Gunther CC, Ramampiaro H, Langaas M, Sandvik AK, Laegreid A (2006). GeneTools--application for functional annotation and statistical hypothesis testing. BMC Bioinformatics.

[B31] Cheadle C, Becker KG, Cho-Chung YS, Nesterova M, Watkins T, Wood W, Prabhu V, Barnes KC (2007). A rapid method for microarray cross platform comparisons using gene expression signatures. Mol Cell Probes.

[B32] Larkin JE, Frank BC, Gavras H, Sultana R, Quackenbush J (2005). Independence and reproducibility across microarray platforms. Nat Methods.

[B33] Bosotti R, Locatelli G, Healy S, Scacheri E, Sartori L, Mercurio C, Calogero R, Isacchi A (2007). Cross platform microarray analysis for robust identification of differentially expressed genes. BMC Bioinformatics.

[B34] Morey JS, Ryan JC, Van Dolah FM (2006). Microarray validation: factors influencing correlation between oligonucleotide microarrays and real-time PCR. Biol Proced Online.

[B35] Wang Y, Barbacioru C, Hyland F, Xiao W, Hunkapiller KL, Blake J, Chan F, Gonzalez C, Zhang L, Samaha RR (2006). Large scale real-time PCR validation on gene expression measurements from two commercial long-oligonucleotide microarrays. BMC Genomics.

[B36] Dallas PB, Gottardo NG, Firth MJ, Beesley AH, Hoffmann K, Terry PA, Freitas JR, Boag JM, Cummings AJ, Kees UR (2005). Gene expression levels assessed by oligonucleotide microarray analysis and quantitative real-time RT-PCR -- how well do they correlate?. BMC Genomics.

[B37] Hofsli E, Thommesen L, Yadetie F, Langaas M, Kusnierczyk W, Falkmer U, Sandvik AK, Laegreid A (2005). Identification of novel growth factor-responsive genes in neuroendocrine gastrointestinal tumour cells. Br J Cancer.

[B38] Halgren RG, Fielden MR, Fong CJ, Zacharewski TR (2001). Assessment of clone identity and sequence fidelity for 1189 IMAGE cDNA clones. Nucleic Acids Res.

[B39] The Norwegian Microarray Consortium. http://www.microarray.no/.

[B40] Lyng H, Badiee A, Svendsrud DH, Hovig E, Myklebost O, Stokke T (2004). Profound influence of microarray scanner characteristics on gene expression ratios: analysis and procedure for correction. BMC Genomics.

[B41] Yang YH, Dudoit S, Luu P, Lin DM, Peng V, Ngai J, Speed TP (2002). Normalization for cDNA microarray data: a robust composite method addressing single and multiple slide systematic variation. Nucl Acids Res.

[B42] Finnish DNA Microarray Centre. http://microarrays.btk.fi/.

[B43] Benjamini Y (1995). Controlling the False Discovery Rate: A Practical and Powerful Approach to Multiple Testing. Journal of the Royal Statistical Society.

[B44] Brazma A, Hingamp P, Quackenbush J, Sherlock G, Spellman P, Stoeckert C, Aach J, Ansorge W, Ball CA, Causton HC, Gaasterland T, Glenisson P, Holstege FC, Kim IF, Markowitz V, Matese JC, Parkinson H, Robinson A, Sarkans U, Schulze-Kremer S, Stewart J, Taylor R, Vilo J, Vingron M (2001). Minimum information about a microarray experiment (MIAME)-toward standards for microarray data. Nat Genet.

[B45] Parkinson H, Sarkans U, Shojatalab M, Abeygunawardena N, Contrino S, Coulson R, Farne A, Lara GG, Holloway E, Kapushesky M, Lilja P, Mukherjee G, Oezcimen A, Rayner T, Rocca-Serra P, Sharma A, Sansone S, Brazma A (2005). ArrayExpress--a public repository for microarray gene expression data at the EBI. Nucleic Acids Res.

