# Supplementary material for: Optimization of cDNA microarrays procedures using criteria that do not rely on external standards
Source: BMC Genomics. 2007 Oct 18;8:377. doi: 10.1186/1471-2164-8-377 (PMC2147032; doi:10.1186/1471-2164-8-377)
Supplement: Additional file 1 — Table S1. Number of differentially expressed genes under different blocking, background correction and levels of filtration. The file shows all combinations of blocking, background correction and levels of filtration together with the estimated numbers of differentially expressed genes with the different combinations. [file 1471-2164-8-377-S1.pdf]

Additional file 1

Table S1: Number of differentially expressed genes under different blocking, background correction and levels of filtration

Background correction	Blocker	Levels of filtration				
		**coarse**	**medium**	**fine**	**uncertainty**	**weight**
**none**	no blocker	122	115	9	121	167
	1000 ng Poly(dA)$_{40-60}$	420	409	355	420	588
	25 ng LNA	497	484	100	546	921
	250 ng LNA	1510	1488	515	1673	2064
	500 ng LNA	983	980	963	995	1162
	1000 ng LNA	1185	1160	420	1360	1766
**Edwards /dampened**	no blocker	28	27	10	27	40
	1000 ng Poly(dA)$_{40-60}$	289	287	209	284	340
	25 ng LNA	11	10	67	11	67
	250 ng LNA	455	451	191	545	588
	500 ng LNA	334	334	810	367	403
	1000 ng LNA	382	381	484	374	431
**Edwards**	no blocker	1	1	11	1	1
	1000 ng Poly(dA)$_{40-60}$	16	15	203	16	16
	25 ng LNA	0	0	65	0	0
	250 ng LNA	68	72	144	66	120
	500 ng LNA	5	4	605	4	1
	1000 ng LNA	2	1	393	2	47
