# Supplementary material for: Optimization of cDNA microarrays procedures using criteria that do not rely on external standards
Source: BMC Genomics. 2007 Oct 18;8:377. doi: 10.1186/1471-2164-8-377 (PMC2147032; doi:10.1186/1471-2164-8-377)
Supplement: Additional file 2 — Table S2. Level of concordance of biological themes represented in the gene list generated with different background correction methods. The file shows all GO terms at hierarchy level 1–3 which was associated with five or more genes(n = 64). [file 1471-2164-8-377-S2.pdf]

Additional file 2

Table S2: Level of concordance of biological themes represented in the gene lists generated with different background correction methods.

GO number	GO term	A	B	AUB	AÚB	BÚA	proportion
GO:0008150	biological_process	724	233	233	491	0	**3.11**
GO:0009987	cellular process	633	204	204	429	0	**3.10**
GO:0008152	metabolic process	459	135	135	324	0	**3.40**
GO:0044238	primary metabolic process	416	122	122	294	0	**3.41**
GO:0044237	cellular metabolic process	416	120	120	296	0	**3.47**
GO:0043170	macromolecule metabolic process	333	103	103	230	0	**3.23**
GO:0065007	biological regulation	315	112	112	203	0	**2.81**
GO:0050789	regulation of biological process	277	96	96	181	0	**2.89**
GO:0032501	multicellular organismal process	249	91	91	158	0	**2.74**
GO:0032502	developmental process	246	91	91	155	0	**2.70**
GO:0050794	regulation of cellular process	231	76	76	155	0	**3.04**
GO:0051179	localization	220	60	60	160	0	**3.67**
GO:0016043	cellular component organization and biogenesis	203	65	65	138	0	**3.12**
GO:0007154	cell communication	202	69	69	133	0	**2.93**
GO:0048856	anatomical structure development	181	70	70	111	0	**2.59**
GO:0007275	multicellular organismal development	181	70	70	111	0	**2.59**
GO:0051234	establishment of localization	179	44	44	135	0	**4.07**
GO:0050896	response to stimulus	165	64	64	101	0	**2.58**
GO:0048869	cellular developmental process	146	52	52	94	0	**2.81**
GO:0019222	regulation of metabolic process	115	36	36	79	0	**3.19**
GO:0048468	cell development	112	38	38	74	0	**2.95**
GO:0009653	anatomical structure morphogenesis	110	45	45	65	0	**2.44**
GO:0009058	biosynthetic process	105	32	32	73	0	**3.28**
GO:0006950	response to stress	101	32	32	69	0	**3.16**
GO:0008283	cell proliferation	75	30	30	45	0	**2.50**
GO:0009605	response to external stimulus	74	28	28	46	0	**2.64**
GO:0022610	biological adhesion	69	35	35	34	0	**1.97**
GO:0007155	cell adhesion	69	35	35	34	0	**1.97**
GO:0042221	response to chemical stimulus	67	28	28	39	0	**2.39**
GO:0009056	catabolic process	65	30	30	35	0	**2.17**
GO:0065008	regulation of biological quality	63	27	27	36	0	**2.33**
GO:0051641	cellular localization	62	13	13	49	0	**4.77**
GO:0016265	death	60	18	18	42	0	**3.33**
GO:0007049	cell cycle	60	13	13	47	0	**4.62**
GO:0033036	macromolecule localization	59	11	11	48	0	**5.36**
GO:0022402	cell cycle process	53	11	11	42	0	**4.82**
GO:0051674	localization of cell	51	17	17	34	0	**3.00**
GO:0006928	cell motility	51	17	17	34	0	**3.00**
GO:0002376	immune system process	48	23	23	25	0	**2.09**
GO:0051239	regulation of multicellular organismal process	45	15	15	30	0	**3.00**
GO:0050877	neurological process	44	12	12	32	0	**3.67**
GO:0006807	nitrogen compound metabolic process	44	13	13	31	0	**3.38**
GO:0040007	growth	42	19	19	23	0	**2.21**
GO:0065009	regulation of a molecular function	41	17	17	24	0	**2.41**
GO:0009719	response to endogenous stimulus	41	9	9	32	0	**4.56**
GO:0006091	generation of precursor metabolites and energy	41	12	12	29	0	**3.42**
GO:0000003	reproduction	38	13	13	25	0	**2.92**
GO:0050793	regulation of developmental process	32	15	15	17	0	**2.13**
GO:0006952	defense response	32	7	7	25	0	**4.57**
GO:0006955	immune response	31	17	17	14	0	**1.82**
GO:0019725	cell homeostasis	30	12	12	18	0	**2.50**
GO:0040008	regulation of growth	26	13	13	13	0	**2.00**
GO:0007610	behavior	25	10	10	15	0	**2.50**
GO:0022414	reproductive process	24	9	9	15	0	**2.67**
GO:0048646	anatomical structure formation	23	13	13	10	0	**1.77**
GO:0016049	cell growth	23	12	12	11	0	**1.92**
GO:0048771	tissue remodeling	22	12	12	10	0	**1.83**
GO:0009790	embryonic development	22	9	9	13	0	**2.44**
GO:0019953	sexual reproduction	19	7	7	12	0	**2.71**
GO:0008015	circulation	18	5	5	13	0	**3.60**
GO:0051301	cell division	17	5	5	12	0	**3.40**
GO:0002520	immune system development	17	7	7	10	0	**2.43**
GO:0009628	response to abiotic stimulus	16	6	6	10	0	**2.67**
GO:0009607	response to biotic stimulus	16	8	8	8	0	**2.00**
GO:0050878	regulation of body fluids	13	8	8	5	0	**1.63**
GO:0001775	cell activation	13	5	5	8	0	**2.60**
GO:0050817	coagulation	11	7	7	4	0	**1.57**

All GO categories associated with more than five genes are shown (n=64).
A: Gene list generated with *no background correction* and *weight based filtering*. **B:** Gene list generated with *dampened Edwards background correction* and *weight based filtering*. **AUB:** number of genes differentially expressed in both gene list A and B. **AÚB:** number of genes differentially expressed in gene list A but not in gene list B. **BÚA:** number of genes differentially expressed in gene list B but not in gene list A. **proportion**: number of genes in A / number of genes in B. The genes were annotated according to UniGen build #163 in GeneTools (http://www.gentools.no).
