# Supplementary material for: Optimization of cDNA microarrays procedures using criteria that do not rely on external standards
Source: BMC Genomics. 2007 Oct 18;8:377. doi: 10.1186/1471-2164-8-377 (PMC2147032; doi:10.1186/1471-2164-8-377)
Supplement: Additional file 3 — Figure S1. Validation of differentially expressed genes by SYBR green-based quantitative real-time PCR. The figure shows fold changes for a few selected genes on the microarry platforms and the correspondence with relative gene expression data from qRT-PCR assays. [file 1471-2164-8-377-S3.pdf]

Additional file 3

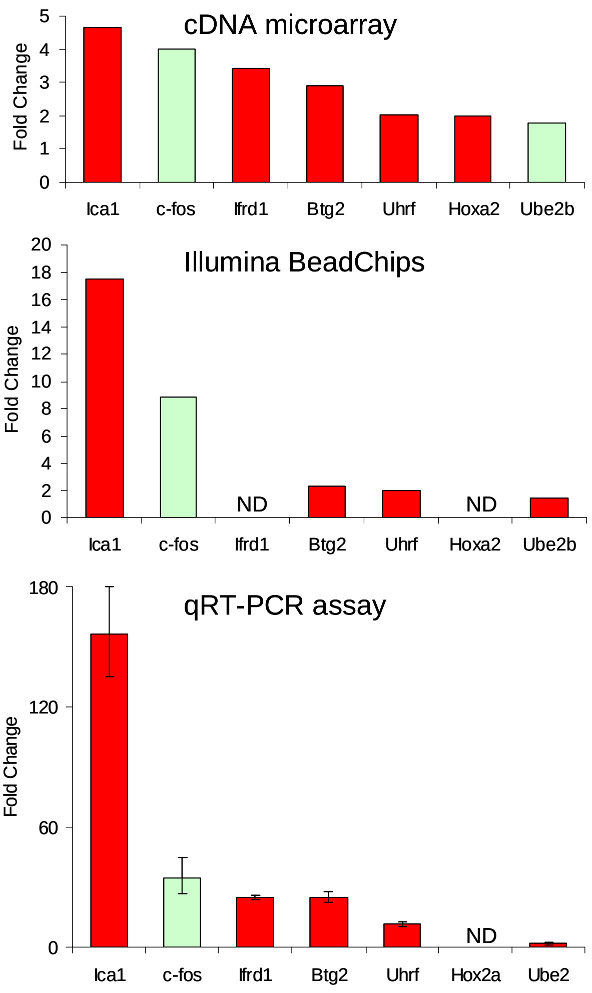

Figure S1. Validation of differentially expressed genes by SYBR green-based quantitative real-time PCR. The figure shows fold changes from a few selected genes on the microarry platforms and relative gene expression data from qRT-PCR assays (lower panel). qRT-PCR was performed in triplicates. Relative gene expressions (fold changes) were calculated by $2^{-\Delta\Delta Ct}$ [1] and mean ± SD values are shown. Red bars indicate genes up-regulated in AR42J cells compared to NRK52E cells. Green bars indicate genes relatively highest expressed in the NRK52E cell line. ND: Not Detectable. More detailed information is given in Additional file 4. PCR primers and qRT-PCR protocol are given in Additional file 5.

1. Livak KJ, Schmittgen TD: **Analysis of relative gene expression data using real-time quantitative PCR and the 2(-Delta Delta C(T)) Method**. *Methods* 2001, **25**(4):402-408.
