# Supplementary material for: Optimization of cDNA microarrays procedures using criteria that do not rely on external standards
Source: BMC Genomics. 2007 Oct 18;8:377. doi: 10.1186/1471-2164-8-377 (PMC2147032; doi:10.1186/1471-2164-8-377)
Supplement: Additional file 4 — Cross platform microarray analysis (Table S3a) and SYBR green-based quantitative real-time PCR (Table S3b). The file shows log2 ratio differences and fold ratios for a few selected genes on the microarray platforms (Table S3a) compared to ΔΔCt values and calculated fold change values from validation by qRT-PCR (Table S3b). [file 1471-2164-8-377-S4.pdf]

Additional file 4

Table S3a: Cross-platform microarray analysis

Gene symbol	cDNA microarray Log (to Base 2)	cDNA microarray Fold Ratio	cDNA microarray AR42J *vs.* NRK52E	Illumina Log (to Base 2)	Illumina Fold Ratio	Illumina AR42J vs NRK52E
Ica1	2.22	4.65	up-regulated	4.13	17.41	up-regulated
c-fos	-2.11	4.32	down-regulated	-3.15	-8.88	down-regulated
Ifrd1	1.79	3.45	up-regulated			
Btg2	1.54	2.91	up-regulated	1.19	2.28	up-regulated
Uhrf	1.02	2.03	up-regulated	0.98	1.97	up-regulated
Hoxa2	1.01	2.02	up-regulated			
Ube2b	-0.88	0.54	dow-regulated	0.52	1.43	up-regulated

Table S3b: SYBR green-based quantitative real-time PCR

Gene symbol	ΔΔCt	$2^{-\Delta\Delta Ct}$ Fold Change	AR42J *vs.* NRK52E	Identifier	UniGene ID	Gene Name
Ica1	-7.29	156.14	up-regulated	UI-R-A0-ax-e-06-0-UI	Rn.1379	Islet cell autoantigen 1
Fos	5.11	0.03	down-regulated	UI-R-C1-lo-f-05-0-UI	Rn.103750	FBJ murine osteosarcoma viral oncogene homolog
Ifrd1	-4.64	24.88	up-regulated	UI-R-A1-ex-e-05-0-UI	Rn.3723	Interferon-related developmental regulator 1
Btg2	-4.65	25.05	up-regulated	UI-R-A1-ew-d-01-0-UI	Rn.27923	B-cell translocation gene 2. anti-proliferative
Uhrf1_mapped	-3.53	11.52	up-regulated	UI-R-E1-fn-h-01-0-UI	Rn.54318	Ubiquitin-like. containing PHD and RING finger domains. 1 (mapped)
Hoxa2	-2.02	4.06*	up-regulated	UI-R-A1-dp-b-10-0-UI	Rn.91077	Homeo box A2
Ube2b	-0.66	1.58	up-regulated	UI-R-E0-cd-c-03-0-UI	Rn.20766	Ubiquitin-conjugating enzyme E2B. RAD6 homolog (S. cerevisiae)

qRT-PCR: Relative gene expression was calculated using the ΔΔCt method where the expression levels were normalized to the level of β-actin expression and AR42J cells were compared to NRK52E cells. Fold chang=$2^{-\Delta\Delta Ct}$. PCR was performed in triplicates in two independent runs. PCR primers and protocol are given in Additional file 5.

**Hoxa2* was excluded from the results due to technical problems with the non RT controls.
