# Supplementary material for: Optimization of cDNA microarrays procedures using criteria that do not rely on external standards
Source: BMC Genomics. 2007 Oct 18;8:377. doi: 10.1186/1471-2164-8-377 (PMC2147032; doi:10.1186/1471-2164-8-377)
Supplement: Additional file 5 — PCR primers (Table S4) and qRT-PCR protocol. The file gives information about the PCR primers and the qRT-PCR protocol used in the study. [file 1471-2164-8-377-S5.pdf]

Additional file 5

Table S4: PCR primers

Gene symbol	GeneBank accession no	Sense 5'- 3'	Antisense 5'- 3'
Ica1	NM_030844	TGGATGAAGGACGTGTCTCA	TGTACCTTCCTGAACTTCTC
c-fos	NM_022197	GTAGAGCAGCTATCTCCTGA	AACGCAGACTTCTCGTCTTC
Ifrd1	NM_019242	GCCAAAGTGGACAAGAGAAA	TACTGCATCCCTGATCCAAG
Btg2	NM_017259	GCACTGACCGATCATTACAA	ATGCGGTAGGACACTTCGTA
Uhrf1_mapped	NM_001008882	ATTTGTAGGAAGCGCCAAAC	ATCCGACAGTTGTTGAGCTG
Hoxa2	NM_0125811	CCCTGGATGAAGGAGAAGAA	GCCATCGGCTATTTCCAG
Ube2b	NM_001008882	AACAACATCATGCAGTGGAA	CAGCATACACATTTGGATGA
β-actin	BC063166	CTGGCTCCTAGCACCATGA	AGCCACCAATCCACACAGA

qRT-PCR protocol.

The same source of total RNA used in the microarray experiments were used for the data validation by qRT-PCR. Total RNA was reverse transcribed by use of ABgene®'s 1st strand Synthesis Kit according to the manufactures instruction. Each SYBR Green real time PCR reaction was performed with 1 × Absolute™ QPCR SYBR® Green Mixes (ABgene UK), 400 nM each of sense (S) and antisense (AS) primers and cDNA equivalent to 62.5 ng of total RNA in a total reaction volume of 25 µl. The qRT-PCR was performed in Stratagene's Mx3000P Real Time PCR system with the following protocol: 15 min at 95 °C, 40 thermal cycles of 15 s at 95 °C, 20 s min at 60 °C, and 40 s at 72 °C. PCR products were verified by dissociation curve analysis to confirm primer specificity. No-reverse transcription controls were performed for each RNA and primer pair. All samples were run in triplicates. Fold induction of gene expression level was estimated by the ΔΔCt-method [1], where the expression levels were normalized to the level of an internal reference gene (β-actin):

Fold Change = $2^{-\Delta\Delta Ct}$ and $\Delta\Delta Ct = (Ct_{GOI} - Ct_{\beta\text{-actin}})_{AR42J} - (Ct_{GOI} - Ct_{\beta\text{-actin}})_{NRK52E}$

1. Livak KJ, Schmittgen TD: **Analysis of relative gene expression data using real-time quantitative PCR and the 2(-Delta Delta C(T)) Method.** *Methods* 2001, **25**(4):402-408.
